# Consolidated Reporting Guidelines for Prognostic and Diagnostic Machine Learning Modeling Studies: Development and Validation

**DOI:** 10.2196/48763

**Published:** 2023-08-31

**Authors:** William Klement, Khaled El Emam

**Affiliations:** 1 University of Ottawa Ottawa, ON Canada; 2 CHEO Research Institute Ottawa, ON Canada

**Keywords:** machine learning, prognostic models, prediction models, reporting guidelines, reproducibility guidelines, diagnostic, prognostic, model evaluation, model training

## Abstract

**Background:**

The reporting of machine learning (ML) prognostic and diagnostic modeling studies is often inadequate, making it difficult to understand and replicate such studies. To address this issue, multiple consensus and expert reporting guidelines for ML studies have been published. However, these guidelines cover different parts of the analytics lifecycle, and individually, none of them provide a complete set of reporting requirements.

**Objective:**

We aimed to consolidate the ML reporting guidelines and checklists in the literature to provide reporting items for prognostic and diagnostic ML in in-silico and shadow mode studies.

**Methods:**

We conducted a literature search that identified 192 unique peer-reviewed English articles that provide guidance and checklists for reporting ML studies. The articles were screened by their title and abstract against a set of 9 inclusion and exclusion criteria. Articles that were filtered through had their quality evaluated by 2 raters using a 9-point checklist constructed from guideline development good practices. The average κ was 0.71 across all quality criteria. The resulting 17 high-quality source papers were defined as having a quality score equal to or higher than the median. The reporting items in these 17 articles were consolidated and screened against a set of 6 inclusion and exclusion criteria. The resulting reporting items were sent to an external group of 11 ML experts for review and updated accordingly. The updated checklist was used to assess the reporting in 6 recent modeling papers in *JMIR AI*. Feedback from the external review and initial validation efforts was used to improve the reporting items.

**Results:**

In total, 37 reporting items were identified and grouped into 5 categories based on the stage of the ML project: defining the study details, defining and collecting the data, modeling methodology, model evaluation, and explainability. None of the 17 source articles covered all the reporting items. The study details and data description reporting items were the most common in the source literature, with explainability and methodology guidance (ie, data preparation and model training) having the least coverage. For instance, a median of 75% of the data description reporting items appeared in each of the 17 high-quality source guidelines, but only a median of 33% of the data explainability reporting items appeared. The highest-quality source articles tended to have more items on reporting study details. Other categories of reporting items were not related to the source article quality. We converted the reporting items into a checklist to support more complete reporting.

**Conclusions:**

Our findings supported the need for a set of consolidated reporting items, given that existing high-quality guidelines and checklists do not individually provide complete coverage. The consolidated set of reporting items is expected to improve the quality and reproducibility of ML modeling studies.

## Introduction

### Background

Prognostic and diagnostic studies that train and apply machine learning (ML) models on health data often fail to adhere to minimal reporting standards [1,2], with inadequate details on model development and evaluation, and fail to fully cover sources of bias [3-5]. Transparent reporting on the development and application of such models is believed to improve reliability, fairness, and usefulness as well as ethical, legal, and regulatory oversight [6].

There are many reporting guidelines and checklists that have been developed for health research [7]. Although a recent evaluation of the use of reporting guidelines by peer reviewers was not able to reach a conclusion on their use and utility [8], one other study found a positive association between reviewer ratings of adherence to reporting guidelines and favorable editorial decisions [9]. Another study reported a significant positive correlation between adherence to reporting guidelines and citations and between adherence to reporting guidelines and publication in higher impact factor journals [10]. Furthermore, there is evidence that the completeness and quality of reporting of research studies is associated with the use of reporting guidelines [11-17].

However, ML modeling studies do not often use reporting guidelines developed for statistical models [18], and reporting deficiencies are being seen in contemporary ML modeling articles [19]. To address this issue, multiple reporting guidelines specific to ML studies have been developed [1,6,20-27]. In general, reporting guideline “inflation” can lead to confusion among authors and peer reviewers regarding the appropriate ones to use [28]. These ML study reporting guidelines overlap but are not the same, with each covering a subset of what can be considered good reporting practice [20]. They each focus on subsets of a typical analytics workflow without being comprehensive. Some guidelines may be nonspecific to health care (eg, DC-Check [26]), and others may omit important aspects of ML modeling methodology (eg, the absence of guidance on model tuning and optimization [21,27]). The existence of multiple guidelines may hinder the adoption of good reporting guidelines in general, as this makes it difficult for researchers to determine the most suitable set of guidelines to use for a particular study and for journal editors to consistently prescribe reporting requirements for authors [20].

### Objectives

In this paper, we consolidated items from current ML reporting guidelines and checklists into a single set. We limited our scope to in-silico studies and those where an ML model is running in shadow mode, as these are necessary first steps in developing ML models that are useful in practice [27]. Given that our items consolidate material from previously published consensus and expert guidelines, they can be used by authors as a checklist to ensure adequate reporting of their studies, by peer reviewers to confirm that important details are included in manuscripts, and by journal editors to ensure that good reporting practices are applied and applied consistently across articles and journals.

## Methods

### Overview

The objective of this study was to identify high-quality ML reporting guidelines and checklists from the literature and consolidate them into a set of reporting items. The approach we followed was informed by recommended practices for conducting scoping reviews [29,30] and developing reporting guidelines [31,32].

The literature search focused on articles that contained a checklist, a flow diagram, or structured text developed to guide authors on the minimum level of detail to include in research papers reporting findings or a specific aspect of research [32].

### Search Criteria

We executed a very broad search query in January 2023 and updated it in June 2023 on PubMed to maximize the capture of published reporting guidelines. The query searched for all articles that contained the term “reporting guideline” with either one of the terms “machine learning” or “artificial intelligence,” that is, “reporting guidelines” AND (“machine learning” OR “artificial intelligence”), limited to English articles published in or after the year 2000. We retrieved 73 articles from the search.

The EQUATOR Network [33] database was searched for articles on “machine learning” and “artificial intelligence” separately. We also adopted an expert-driven approach by curating recent review articles that presented reporting guidelines, reproducibility guidelines, reviews of guidelines, or critique articles on ML practices in medical or biological studies. Recursively, we also reviewed their respective references that reported informative items of the strengths or weaknesses of ML-based studies in medicine. This enabled us to identify an additional 137 articles.

After removing duplicates, there were 192 articles remaining.

### Article Inclusion and Exclusion Criteria

The titles and abstracts of the 192 identified articles were reviewed by one of the authors (WK) and screened according to the inclusion and exclusion criteria presented in Textbox 1.

**Figure 1:**
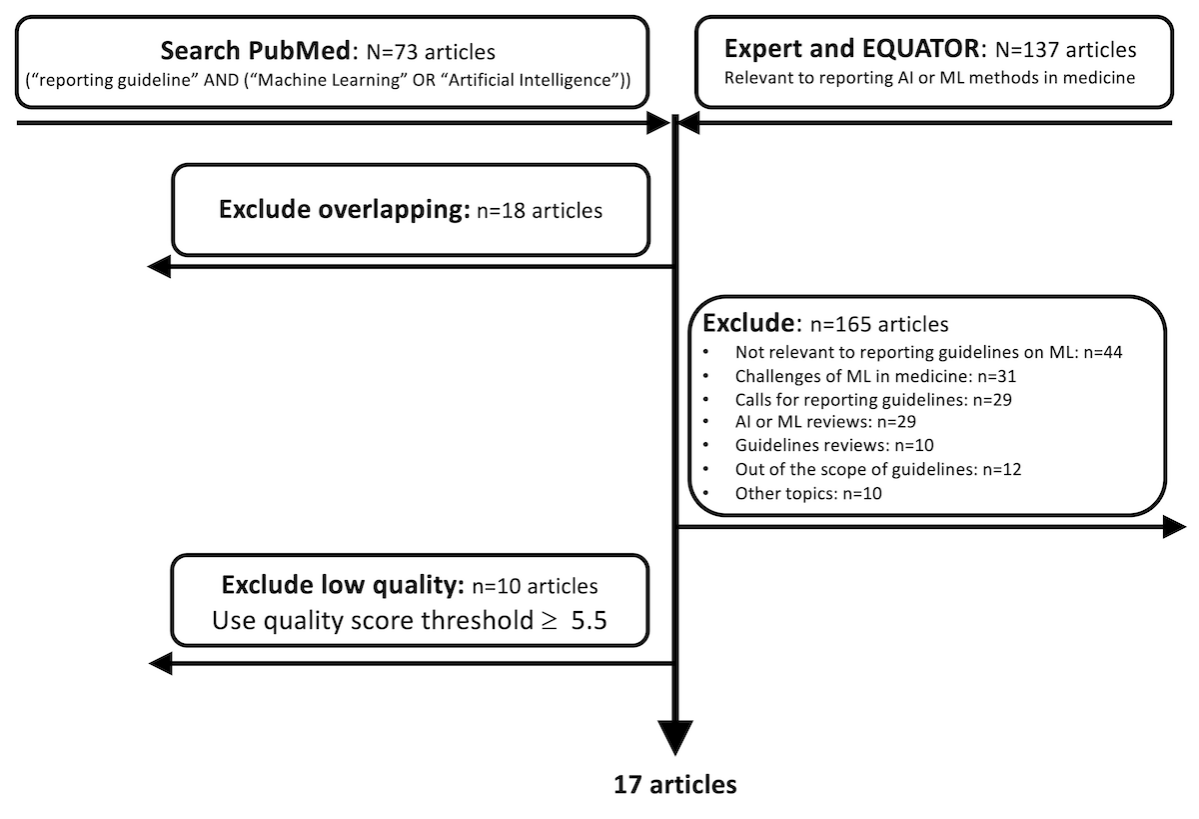
PRISMA (Preferred Reporting Items for Systematic Reviews and Meta-Analyses) diagram for machine learning (ML) reporting guidelines search. AI: artificial intelligence.

As illustrated in the PRISMA (Preferred Reporting Items for Systematic Reviews and Meta-Analyses) diagram [36] in Figure 1, the selection criteria resulted in 27 articles. These were then subjected to a quality assessment, as described in the following section.

**Inclusion and exclusion criteria.**

**Inclusion criteria**

- The article was peer reviewed in journals or conferences (ie, no preprints or technical reports were included) unless the article was initially expert curated or recommended.
- The article met the definition of a reporting guideline. We generalized the definition in the study by Schlussel et al [32] to include articles that were not focused on medical research because there are machine learning reporting guidelines that are domain agnostic that still contain useful guidance.
- The reporting guideline article must be specific to machine learning models (ie, guidelines that were specific to statistical prognostic or diagnostic models were excluded).
- The reporting guideline must be new, an update, or an extension.
- Articles that were exclusive to certain types of data, such as images, unstructured text, and genomic sequences, were excluded. The default type of data assumed in this consolidated guideline is structured data. Although most health data are unstructured [34] and have significant value when analyzed [35], an examination of all articles (excluding editorials) published in *JMIR AI* at the time of writing indicated that 23% involved the analysis of structured data, 11% involved the analysis of images, and 35% involved text. Therefore, to the extent that these numbers are reflective of the current published research in medical ML, the focus on structured data is still relevant to at least one-fifth of that body of work, especially given that reporting guidelines and checklists are targeted at improving the reporting of *published* articles. Articles covering structured data *and* other additional data modalities were included (per the item inclusion and exclusion criteria described in the *Item Inclusion and Exclusion Criteria* section, the reporting items that were not on structured data in these multimodal guideline articles were removed).

**Exclusion criteria**

- Articles that review or evaluate existing reporting guidelines.
- Articles that call for or make the case for reporting guidelines or better reporting guidelines.
- Articles that describe or report on the use of ML in a particular specialty.
- Articles that describe methods for developing reporting guidelines.

### Quality Assessment

We constructed a checklist to evaluate the quality of reporting guidelines and checklists based on the recommendations in the literature on good practices for guideline development [31,32,37,38]. We only considered criteria that were relevant for our purposes (eg, whether a reporting guideline had a website was not considered a quality criterion that was relevant for our purposes). The quality assessment criteria are listed in Table 1.

**Table 1:** Quality assessment criteria for the guidelines and checklists identified in the literature. The response categories used were “yes,” “no,” “N/A,” and “cannot tell.” The average score is taken across all 27 articles that were evaluated.

Quality criteria	Value, κ (95% CI)	Score, mean (SD)
1. The need for guidance and reporting checklist is clearly defined	1^a^	1.00 (0.00)
2. There was a literature review (either in the article or cited) to identify previous relevant guidance and requirements	1^a^	0.98 (0.10)
3. The methods for that previous literature review have been described	0.68 (0.67 to 0.69)	0.37 (0.45)
4. Inclusion criteria for the reporting items in the preliminary list were defined	−0.054 (−0.52 to −.05)^b^	0.94 (0.16)
5. A Delphi exercise was performed to define and narrow down the initial reporting item list	0.91 (0.9 to 0.91)	0.28 (0.45)
6. The Delphi group was representative (includes, eg, academics, journal editors, policy makers, industry, funders, patients, regulators, REB^c^ members, medical writing professionals, librarians)	0.91 (0.9 to 0.91)	0.28 (0.45)
7. A face-to-face meeting was conducted to reach consensus on the items and their definitions among the expert group (virtual meetings that enabled discussions of items were also considered acceptable)	0.53 (0.51 to 0.54)^d^	0.81 (0.34)
8. The checklist was pilot tested	0.4 (0.39 to 0.41)^d^	0.44 (0.42)
9. The checklist and its development methodology were published in a peer-reviewed journal	1^a^	0.98 (0.10)

^a^Perfect agreement between the raters.

^b^A known behavior of κ is that when the expected agreement is already high, the κ value can be quite low [39], which is the case here. Alternative statistics have been proposed [40], but they have less interpretation guidance. Therefore, we reported the proportion of positive agreement (equal to 0.94) and the proportion of negative agreement (equal to 0), as suggested in these circumstances [41]. The 2 raters had an almost perfect positive agreement.

^c^REB: Research Ethics Board.

^d^A third rater (second coauthor of this paper: KEE) reconciled the differences between the 2 raters to obtain a final score on this criterion.

Two independent reviewers, one coauthor (WK) and an independent reviewer, separately evaluated each of the identified 27 articles. The κ statistic was used to evaluate the interrater agreement of the score for each quality criterion, and the results are shown in Table 1. The average κ across all criteria was 0.71. Values equal to and above 0.61 are considered to be moderate [42].

The use of a Delphi method to narrow down the reporting items was not used very often (criteria 5 and 6), and the literature review that was performed was not always clearly documented (criterion 3). Many guideline development efforts have used a form of expert meeting to review the reporting items (criterion 7), although we did not require these to be face to face. Pilot testing of reporting guidelines was performed in less than half the time (criterion 8).

We considered an article to be of high quality if at least 61% of the responses were “yes” when each criterion score was averaged across the 2 raters (after reconciliation, where relevant). This threshold also coincides with the median of the quality score. This threshold resulted in 17 articles that were assigned a quality score≥5.5. The scores for each of the 27 articles and whether they scored high or low are shown in Table 2.

As a sensitivity analysis, if a higher threshold score of 7 is used (77% “yes” responses on the quality criteria; this is the mean value), this would have resulted in the exclusion of only 1 item from our final list. Therefore, there was little sensitivity in the 61% to 77% threshold range.

**Table 2:** The 27 reporting guidelines articles selected for consolidation. The level of article quality (high or low) was determined by reference to the threshold ≥5.5 on the average quality score between the 2 independent raters.

Label	Article title	Reference	Year	Quality score (overall mean 6, SD 1.66; median 5.5, IQR 2)	Quality assessment
A01	Reporting guideline for the early-stage clinical evaluation of decision support systems driven by artificial intelligence: DECIDE-AI	[27]	2022	8.5	High
A02	Machine Learning Methods in Health Economics and Outcomes Research—The PALISADE Checklist: A Good Practices Report of an ISPOR Task Force	[22]	2022	4.5	Low
A03	Nuclear Medicine and Artificial Intelligence: Best Practices for Evaluation (the RELAINCE Guidelines)	[43]	2022	5.5	High
A04	DC-Check: A Data-Centric AI checklist to guide the development of reliable machine learning systems	[26]	2022	5	Low
A05	Critical appraisal of artificial intelligence-based prediction models for cardiovascular disease	[44]	2022	4	
A06	DOME: recommendations for supervised machine learning validation in biology	[45]	2021	5.5	High
A07	Presenting artificial intelligence, deep learning, and machine learning studies to clinicians and healthcare stakeholders: an introductory reference with a guideline and a Clinical AI Research (CAIR) checklist proposal	[21]	2021	5	Low
A08	Low adherence to existing model reporting guidelines by commonly used clinical prediction models	[20]	2021	7	High
A09	The need to separate the wheat from the chaff in medical informatics	[1]	2021	4.5	Low
A10	Review of study reporting guidelines for clinical studies using artificial intelligence in healthcare	[4]	2021	5.5	High
A11	Reporting guidelines for clinical trial reports for interventions involving artificial intelligence: the CONSORT-AI extension	[46]	2020	9	High
A12	Best practices for authors of healthcare-related artificial intelligence manuscripts	[47]	2020	3.5	Low
A13	Guidelines for clinical trial protocols for interventions involving artificial intelligence: the SPIRIT-AI extension	[3]	2020	9	High
A14	Machine learning and artificial intelligence research for patient benefit: 20 critical questions on transparency, replicability, ethics, and effectiveness	[48]	2020	5	Low
A15	Minimum information about clinical artificial intelligence modeling: the MI-CLAIM checklist	[49]	2020	5	Low
A16	Proposed Requirements for Cardiovascular Imaging-Related Machine Learning Evaluation (PRIME): A Checklist: Reviewed by the American College of Cardiology Healthcare Innovation Council	[50]	2020	5.5	High
A17	MINIMAR (MINimum Information for Medical AI Reporting): Developing reporting standards for artificial intelligence in health care	[51]	2020	4	Low
A18a^a^	PROBAST: A Tool to Assess the Risk of Bias and Applicability of Prediction Model Studies	[52]	2019	9	High
A18b^a^	PROBAST: A Tool to Assess Risk of Bias and Applicability of Prediction Model Studies: Explanation and Elaboration	[53]	2019	9	High
A19	Guidelines for Developing and Reporting Machine Learning Predictive Models in Biomedical Research: A Multidisciplinary View	[54]	2016	7	High
A20	Transparent Reporting of a multivariable prediction model for Individual Prognosis Or Diagnosis (TRIPOD): The TRIPOD Statement	[55]	2015	7	High
A21	Critical Appraisal and Data Extraction for Systematic Reviews of Prediction Modelling Studies: The CHARMS Checklist	[56]	2014	6.5	High
A22	Towards better clinical prediction models: seven steps for development and an ABCD for validation	[57]	2014	5.5	High
A23	Improving Reproducibility in Machine Learning Research	[58]	2020	6	High
A24	The AIMe registry for artificial intelligence in biomedical research	[59]	2021	6	High
A25	Recommendations for reporting machine learning analyses in clinical research	[60]	2020	5	Low
A26	CheckList for EvaluAtion of Radiomics research (CLEAR): a step-by-step reporting guideline for authors and reviewers endorsed by ESR and EuSoMII	[61]	2023	8	High
A27	Artificial intelligence in dental research: Checklist for authors, reviewers, readers	[62]	2021	8.5	High

^a^There are 27 articles in total with A18a and A18b presenting the same content.

### Item Inclusion and Exclusion Criteria

Reporting items were identified in the 17 articles that made it through the quality assessment. Items in these articles were excluded if they met any of the following criteria:

- The items were not within our defined scope of in-silico and shadow mode studies. For example, the items covered ML model implementation and deployment details, such as how models affected clinical pathways and the requirements for the training and education of clinicians. These may have appeared in articles that discuss ML model evaluation in practice and model deployment exclusively or as part of the entire model development and implementation life cycle.
- They did not pertain to structured data. For example, the items pertained to images or text. This sometimes occurred in articles that covered multiple data modalities.
- The items described detailed technical reproducibility requirements, such as guidelines for organizing and documenting code, preparation of virtual machines and Docker containers, and documentation of computational environments were excluded.
- The items pertained to the methodology used for the collection of the training data used in the study. The data sets used in model training may have been collected using different prospective or retrospective designs that may have been controlled. There are additional reporting requirements specific to these designs, such as randomized controlled trials, which would complement the guidelines described in this paper [3,27,46,63].
- Items that were specific to the reporting of theoretical results and mathematical proofs pertaining to ML models were excluded.
- The reporting guidelines presented in this paper are intended to reflect best practices today rather than ideal practices that would be challenging for contemporary researchers to meet (eg, because the methodological issue remains in the formative stages of exploration and development). Any reporting items that were deemed formative were excluded.

Note that the abovementioned exclusions pertain to items and not articles. For example, guideline articles such as DECIDE-AI (Developmental and Exploratory Clinical Investigations of Decision support systems driven by Artificial Intelligence), CONSORT-AI (Consolidated Standards of Reporting Trials–Artificial Intelligence), and SPIRIT-AI (Standard Protocol Items: Recommendations for Interventional Trials–Artificial Intelligence) discuss the use of clinical trial designs to evaluate ML models. The specific reporting items that pertain to the conduct of clinical trials were not considered, but other items that were specific to the modeling task were included in our review.

This set of items was converted into a checklist that can be used to ensure that all relevant information has been reported.

### Expert Review of Reporting Items or the Checklist

The resultant checklist was sent to 11 members of the *JMIR AI* editorial board for an independent review. They were asked to comment on the clarity of the definitions and levels of granularity of the reporting items. They were also invited to identify any significant omissions that were not covered by the consolidated list and any challenges that they foresee in its application. The feedback from the editorial board was incorporated into a revision of the reporting items and checklist in this paper.

### Initial Validation

Six prognostic or diagnostic ML studies that were published in *JMIR AI* were identified, and the resulting items were used to assess whether the authors reported the specific details. The perspective of this assessment was as a reviewer and was not intended to score the papers on adherence to our reporting items. This was performed by one of the authors (KEE). The result of that effort was used to further revise the item definitions, item descriptions, and checklist to ensure that they can be applied in practice.

### Presenting the Consolidated Reporting Items or Checklist

During the presentation of the consolidated reporting items and checklist, a number of general principles were followed in their presentation to ensure that they would be practically useful and within our scope:

- Some of the source articles that we draw from provide methodology recommendations (ie, explanation and elaboration of the items). We limited our focus to the information that needs to be reported only while keeping methodology recommendations to a minimum (eg, to illustrate a type of reporting). Methodology guidance is outside the scope of this paper.
- Given that this is a consolidation, new items were not introduced. Therefore, all the items are derived from existing recommendations to ensure consistency with the literature and the objectives of the study.
- Our consolidated reporting items and checklist were categorized into 5 groups according to an analytics workflow consistent with standard process models for data mining [64], including describing the study details and problem being addressed, the data, the modeling approach, performance evaluation, and model interpretation.

## Results

### Overview

We identified 37 reporting items from 17 high-quality reporting guideline articles. The articles were published between 2014 and 2023, and most articles were published in the last 3 years.

The percentage of articles that covered each of the categories is shown in Table 3. We see that the median percentage of “data description” articles that appeared in source articles was 75%, but only a median of 33% of the “model explainability” items appeared in a source article. This table indicates the coverage of each category in the ML reporting guidelines literature and illustrates the importance of developing a consolidated set of reporting items across this body of work.

**Table 3:** Percentages of reporting items discussed in the 17 high-quality guidelines articles per each of the categories. The categories are ordered from high to low by the median percentage of items per source article.

Description	Values, median (range; %)^a^	Values, mean (SD; %)^a^
Data description	75 (50-100)	74 (14)
Study details	70 (10-100)	63 (29)
Model evaluation	67 (17-83)	63 (17)
Methodology	40 (20-90)	44 (18)
Model explainability	33 (0-100)	39 (32)

^a^These values are rounded to the nearest integer.

Although the items assume that the observations pertain to patients, they can also pertain to providers or administrators, depending on the study. Furthermore, the following order of categories follows a theoretical workflow sequence for a study; however, this may differ from how the information is actually presented in an article or a report.

The reporting items and checklist are described in terms of the information that needs to be documented in a study report. They are defined assuming a single ML model that is being developed and evaluated, but in practice, multiple models may be part of the same study, and the items should be generalized accordingly.

In subsequent sections, we present reporting items grouped into the 5 categories.

### Category 1: Study Details

#### Overview

A mapping of the category 1 items to the articles is presented in Table 4. Here, we can see that the article with the highest coverage for this category is A13, with all items included (100%), and articles A23 and A06 have the smallest coverage where only 1 item from this category is mentioned (1/10, 10%). In addition, item 1.1 is mentioned the most in 88% (15/17) of the source articles and item 1.6 is mentioned the least in only 41% (7/17) of the source articles. Article A13 had a high-quality score, whereas articles A23 and A06 had quality scores that were lower and closer to the median threshold.

**Table 4:** Articles that discuss reporting items in category 1 (study details; n=17 articles)^a^.

Articles	Quality score	Reporting items											Total (n=10 items), n (%)
		1.1 (n=15, 88%)	1.9 (n=13, 76%)	1.8 (n=12, 71%)	1.3 (n=11, 65%)	1.5 (n=11, 65%)	1.2 (n=10, 59%)	1.4 (n=10, 59%)	1.7 (n=10, 59%)	1.10 (n=8, 47%)	1.6 (n=7, 41%)		
A13	9	✓	✓	✓	✓	✓	✓	✓	✓	✓	✓	10 (100)	
A18a+b	9	✓	✓	✓	✓	✓	✓	✓	✓		✓	9 (90)	
A27	8.5	✓	✓	✓	✓	✓	✓	✓	✓	✓		9 (90)	
A19	7	✓	✓	✓	✓	✓		✓	✓	✓	✓	9 (90)	
A10	5.5	✓	✓	✓	✓	✓	✓	✓	✓		✓	9 (90)	
A11	9	✓	✓	✓	✓		✓		✓	✓	✓	8 (80)	
A26	8	✓	✓		✓	✓	✓	✓		✓	✓	8 (80)	
A20	7	✓	✓	✓	✓	✓	✓	✓	✓			8 (80)	
A01	8.5	✓		✓	✓	✓	✓		✓	✓		7 (70)	
A21	6.5	✓	✓	✓	✓		✓		✓			6 (60)	
A03	5.5	✓	✓	✓		✓			✓		✓	6 (60)	
A16	5.5	✓	✓	✓		✓				✓		5 (50)	
A22	5.5	✓			✓	✓	✓	✓				5 (50)	
A24	6	✓	✓					✓		✓		4 (40)	
A08	7	✓		✓								2 (20)	
A23	6							✓				1 (10)	
A06	5.5		✓									1 (10)	

^a^The articles are ordered vertically by the percentage of items that are covered per article. The items are ordered horizontally by the percentage of items that are covered by a particular article. The quality score for each article is also shown in the second column.

A description of the items in this category is presented in subsequent sections.

#### Item 1.1: The Medical and Clinical Task of Interest

The focus is on tasks that can be characterized as diagnostic, where they estimate the presence of disease or condition or prognostic, which forecasts the occurrence of a specific future event.

#### Item 1.2: The Research Question

The outcomes of interest should be defined. Present factors and insights into what is involved in determining the outcome or in estimating the risk of the end point in the context of the medical and clinical task described earlier [57]. This helps to clarify the relevance, importance, challenges, and contributions of the proposed analysis.

#### Item 1.3: Current Medical and Clinical Practice

To effectively propose a diagnostic and prognostic model, the current practice and standard of care at the relevant institution or community in general should be understood. To this extent, describe how diagnosis and prognosis are currently established, at what stage of disease, and toward what end point.

#### Item 1.4: The Known Predictors and Confounders to What Is Being Predicted or Diagnosed

Predictors should be specified with justifications (eg, from the literature). Comorbidities, interventions, and administered treatments are a few of many possible confounders that may be involved in diagnosis or prognosis. Understanding the confounders will enhance the validity of the study and clarify its limitations.

It is also important to ensure that none of the covariates are a proxy for the outcome and would not be available at the time of decision-making, as that would negatively impact the value of the model. For example, if a covariate is an indicator of prescribing a drug indicated for a disease and the model is predicting whether a patient has the disease, then if the covariate is known, we would know the outcome (ie, the model is likely not useful).

#### Item 1.5: The Overall Study Design

The training, validation, and test data may have been collected through, for example, observational methods, case-control studies, cohort studies, and population studies. They may also be prospective or retrospective studies. Key details of the study design that resulted in the data should be described.

#### Item 1.6: The Medical Institutional Settings

The setting could be, for example, a hospital, nursing home, or epidemiological center, where the modeling study is conducted, where the ML model will be used (usually these are the same, but not always), and where the data have been or are being collected.

#### Item 1.7: The Target Patient Population

This is the population that the model is intended to generalize to.

#### Item 1.8: The Intended Use of the ML Model

Explain the intended use of the ML model as part of the clinical pathway. Describe its purpose and its respective users (eg, medical staff, technicians, patients, and the public). If applicable, clarify how it may be integrated into practice [1,2]. Set out the expertise expected of intended users of the ML model.

#### Item 1.9: Existing Model Performance Benchmarks for This Task

Describe preexisting evidence of using ML methods applied to the medical and clinical task described earlier. If available, summarize the existing model performance (such as the area under the receiver operating characteristic curve results) to establish a benchmark performance for comparison. Where the ground truth is required to interpret the evaluation results, how that ground truth was defined needs to be described.

#### Item 1.10: Ethical and Other Regulatory Approvals Obtained

Standard reporting requirements include the consent of participants, approvals of ethical agencies, regulatory compliance statements and declarations, certifications required, funding, and conflicts of interest [22,27,44,48,65].

### Category 2: The Data

#### Overview

There is a need to assess the quality and representativeness of data in the context of the study problem, as relevant to the question and outcomes described in category 1. A mapping of the category 2 items to the articles is presented in Table 5.

**Table 5:** Articles that discuss reporting items in category 2 (the data; n=17 articles)^a^.

Articles	Quality score	Reporting items									Total (n=8 items), n (%)
		2.4 (n=16, 94%)	2.7 (n=16, 94%)	2.5 (n=15, 88%)	2.6 (n=14, 82%)	2.8 (n=14, 82%)	2.1 (n=11, 65%)	2.2 (n=11, 65%)	2.3 (n=4, 24%)		
A21	6.5	✓	✓	✓	✓	✓	✓	✓	✓	8 (100)	
A13	9	✓	✓	✓	✓	✓	✓	✓		7 (88)	
A01	8.5	✓	✓	✓	✓	✓	✓	✓		7 (88)	
A27	8.5	✓	✓	✓	✓	✓	✓	✓		7 (88)	
A03	5.5	✓	✓	✓	✓	✓		✓	✓	7 (88)	
A10	5.5	✓	✓	✓	✓	✓	✓	✓		7 (88)	
A11	9	✓	✓	✓	✓		✓	✓		6 (75)	
A18a+b	9	✓	✓	✓	✓		✓		✓	6 (75)	
A26	8	✓	✓	✓	✓	✓	✓			6 (75)	
A19	7	✓	✓	✓		✓	✓	✓		6 (75)	
A23	6	✓	✓	✓	✓	✓		✓		6 (75)	
A24	6		✓	✓		✓		✓	✓	5 (63)	
A06	5.5	✓	✓	✓	✓	✓				5 (63)	
A16	5.5	✓	✓	✓	✓	✓				5 (63)	
A22	5.5	✓	✓		✓		✓	✓		5 (63)	
A08	7	✓		✓	✓	✓				4 (50)	
A20	7	✓	✓			✓	✓			4 (50)	

^a^The articles are ordered vertically by the percentage of items that are covered per article. The items are ordered horizontally by the percentage of items that are covered by a particular article. The quality score for each article is also shown in the second column.

#### Item 2.1: Inclusion and Exclusion Criteria for the Patient Cohort

This item details the patient inclusion and exclusion criteria, along with any treatments administered. Additional details on how the cohort is representative of the population would be provided here.

#### Item 2.2: Methods of Data Collection

Data collection methods can be characterized as retrospective or prospective and by the timeliness and frequency of collection as per the study design (eg, cross-sectional at a single time point or longitudinal at multiple time points). Where relevant, characterize the patient burden of data collection and any data collection challenges. For example, tissue biopsy collection is considered more intrusive than drawing blood samples from a clinical point of view. Intuitively, such issues will impact the availability of data and will influence the reproducibility of results.

#### Item 2.3: Bias Introduced Due to the Method of Data Collection Used

Potential error or bias may be introduced by the method of collection because clinical practice can lead to undesired bias. For instance, the collection of tissue biopsies from cancer patients can be highly associated with the presence of tumors because a biopsy is collected only when the patient is highly suspected of having a tumor. Conversely, obtaining a biopsy of a tumor in an advanced stage of cancer may trigger undesired tumor growth. Consequently, a higher or lower degree of association between sample collection and the presence of tumors can potentially be observed.

#### Item 2.4: Data Characteristics

Data characteristics include the number of records or data points (this number may differ from individuals included in the study if there are multiple records per individual, as in a longitudinal data set); dimension (the number of features); type (categorical, ordinal, continuous, and text); level of missingness for each feature (by class for categorical variables); range of respective values; cardinality (for categorical features); units; and relevant mappings and encodings. Where observations were manually classified or labeled, the number of annotators should be specified.

In addition, the authors should report empirical characteristics (or descriptive statistics) on patient data (including demographics and end point outcome characteristics). This can include proportions, central tendency, and variation.

#### Item 2.5: Methods of Data Transformations and Preprocessing Applied

Transformations include those to normalize or standardize features, for the calculation of derived features, for the use of discretization and cutoffs, and for any embedding layers constructed to handle high-cardinality variables. These should be described including the order in which the transformations are applied where relevant. It should be clarified whether data-informed transformations (eg, dichotomization around a median cutoff) were performed on the training data only, as otherwise data leakage may affect model evaluation results.

#### Item 2.6: Known Quality Issues With the Data

Quality issues may be indicated by the level of missingness, known systematic or random biases, and interobservation dependencies. Manual data collection steps can introduce quality issues such as mislabeling and poor interobserver agreement for manually coded variables. Quality may also be relative to the analytical method used. For example, if there is an assumption of a normal distribution by a particular analytic technique and the data are heavily skewed, then arguably the data have poor quality for this particular study. Where relevant, quality metrics should be provided to quantify such data problems.

#### Item 2.7: Sample Size Calculation

To achieve specific performance and stability, the size of the training data set must be sufficiently large. The method of calculating the sample size along with any assumptions made to support the calculation should be described.

#### Item 2.8: Data Availability

Funding agencies are increasingly requiring that data sets be shared more broadly [66-68]. Information about data availability would be part of a study’s data management and data sharing plan for newly generated data sets [68]. For data that already exist in repositories or from specific data custodians (such as national statistical agencies), information on how to access or request the data should be provided.

### Category 3: Methodology

#### Overview

Reporting items in this category are concerned with methodological strategies used during the development of an ML model. A mapping of the category 3 items to the articles is presented in Table 6.

**Table 6:** Articles that discuss reporting items in category 3 (methodology; n=17 articles)^a^.

Articles	Quality score	Reporting items											Total (n=10 items), n (%)
		3.8 (n=14, 82%)	3.1 (n=13, 76%)	3.9 (n=12, 71%)	3.10 (n=11, 65%)	3.2 (n=6, 35%)	3.5 (n=5, 29%)	3.7 (n=5, 29%)	3.3 (n=3, 18%)	3.4 (n=3, 18%)	3.6 (n=3, 18%)		
A16	5.5	✓	✓	✓	^b^	✓	✓	✓	✓	✓	✓	9 (90)	
A26	8	✓	✓	✓		✓	✓	✓	✓			7 (70)	
A27	8.5	✓	✓	✓	✓	✓	✓					6 (60)	
A19	7	✓	✓	✓	✓			v		✓		6 (60)	
A08	7	✓	✓	✓	✓					✓		5 (50)	
A10	5.5	✓	✓	✓	✓		✓					5 (50)	
A21	6.5	✓	✓		✓	✓						4 (40)	
A23	6	✓		✓		✓					✓	4 (40)	
A24	6			✓			✓	✓			✓	4 (40)	
A03	5.5	✓		✓	✓			✓				4 (40)	
A06	5.5	✓		✓		✓			✓			4 (40)	
A11	9		✓	✓	✓							3 (30)	
A13	9		✓	✓	✓							3 (30)	
A01	8.5	✓	✓		✓							3 (30)	
A20	7	✓	✓		✓							3 (30)	
A22	5.5	✓	✓		✓							3 (30)	
A18a+b	9	✓	✓									2 (20)	

^a^The articles are ordered vertically by the percentage of items that are covered per article. The items are ordered horizontally by the percentage of items that are covered by a particular article. The quality score for each article is also shown in the second column.

#### Reporting on Activities Performed Before Training the ML Model

The items in the subsequent sections pertain to activities that would be performed during a data preprocessing stage. A particular general concern is data leakage, in which spurious relationships are induced between the covariates and the outcomes.

#### Item 3.1: Strategies for Handling Missing Data

An ML algorithm may or may not tolerate missingness. Methods for dealing with missingness may remove data records that have missing data (complete case analysis); however, this reduces the sample size and may introduce bias. Alternatively, the detrimental effect of missing data can be minimized by selecting features that avoid it or by imputing the missing values needed for the analysis [69]. Each approach has advantages and disadvantages [70-72]. Reporting the strategies used in the study will enable the assessment of how missingness impacts the representativeness of concepts in the data.

#### Item 3.2: Strategies for Addressing Class Imbalance

Class imbalance is often common in medical data, and in many cases, it can be severe (ie, the minority class is <10% of the data [73]). Reporting the presence and magnitude of class imbalance and how and when it was dealt with is important to allow for the proper evaluation of the study methodology and results.

#### Item 3.3: Strategies for Reducing Dimensionality of Data

If used in the study, describe methods that optimize the number of features, such as principal component analysis, and feature selection. Feature selection should be performed on the training data, and the resulting features should be reused in the test data [74].

#### Item 3.4: Strategies for Handling Outliers

Sources of outliers in the data include input errors, corruption, and true outlier measurements. Many methods have been proposed to detect and deal with outliers. Some ML algorithms can deal with outliers by assigning lower weights to them, but sometimes, outliers are removed from the training data. These strategies should be reported.

#### Item 3.5: Strategies for Data Augmentation

In some cases, synthetic data are generated to increase the size of the available data for reasons ranging from class balancing, increasing data diversity, to intentionally adding noise—possibly to counter overfitting. If used, it is necessary to discuss the objectives, rationale, methods, and impact on the ML algorithm used. Furthermore, reporting the potential impact of augmentation on prediction performance and interpretation is recommended.

#### Item 3.6: Strategies for Model Pretraining

Transfer learning involves transferring knowledge from training the model on another data set and then continuing to train the resulting model on the data at hand to solve the new problem. For instance, a model may be pretrained on a completely different data set, or it can be pretrained on different training data collected as part of this study. In either situation, report on the rationale, methodology, and similarity between data sets and results.

#### Reporting on Model Training

The following items pertain to activities involved in the training of an ML model.

#### Item 3.7: The Rationale for Selecting the ML Algorithm

The rationale for selecting the algorithms should be presented, especially when compared with alternatives. Discuss the rationale and justification for why the ML model is useful for solving the problem at hand rather than a traditional statistical model (eg, why is a logistic regression model not sufficient). This may be justified by the results from previous studies.

The choice of algorithms can also be based on how well their requirements are met. For example, an artificial neural network will require a substantial number of training examples and may not be suited for problems with limited data.

#### Item 3.8: The Method of Evaluating Model Performance During Training

To avoid optimism in model error estimates, appropriate partitioning, cross-validation, or bootstrapping methods can be used. The parameters (eg, proportion of train or test split, number of bootstrap iterations, or number of folds) need to be reported.

#### Item 3.9: The Method Used for Hyperparameter Tuning

Define the range of possible values evaluated for all hyperparameters, present the method used to select or optimize hyperparameter values (eg, grid search or Bayesian optimization), and describe any cross-validation strategy used during the tuning phase. In addition, define the optimization metric used for model tuning (eg, log loss and mean squared error). It is important to describe the behavior of the performance metrics and to clarify and motivate their use and interpretability in the context of the study.

#### Item 3.10: Model’s Output Adjustments

Models can still be adjusted after training but before testing. For example, a classification threshold can be adjusted and the classification scores may be calibrated. Report on whether such modifications took place and describe how and why they were made. For example, if the model was designed to produce a pseudoprobability of classification, then calibration may be necessary to ensure proper probability estimates (eg, when using ensemble learning or balancing otherwise imbalanced data). In this case, details on the method of calibration will support the understanding of the results.

### Category 4: Model Evaluation

#### Overview

This category is focused on ML model performance evaluation and its reporting. A mapping of the category 4 items to the articles is presented in Table 7.

**Table 7:** Articles that discuss reporting items in category 4 (model evaluation; n=17 articles)^a^.

Articles	Quality score	Reporting items							Total (n=6 items), n (%)
		4.3 (n=16, 94%)	4.5 (n=16, 94%)	4.1 (n=13, 76%)	4.4 (n=10, 59%)	4.2 (n=7, 41%)	4.6 (n=2, 12%)		
A27	8.5	✓	✓	✓	✓	✓		5 (83)	
A19	7	✓	✓	✓	✓	✓		5 (83)	
A03	5.5	✓	✓	✓	✓		✓	5 (83)	
A16	5.5	✓	✓	✓	✓	✓		5 (83)	
A13	9	✓	✓			✓	✓	4 (67)	
A26	8	✓	✓	✓	✓			4 (67)	
A08	7	✓	✓	✓	✓			4 (67)	
A23	6	✓	✓	✓	✓			4 (67)	
A24	6	✓	✓	✓	✓			4 (67)	
A06	5.5	✓	✓	✓	✓			4 (67)	
A10	5.5	✓	✓	✓	✓			4 (67)	
A18a+b	9	✓	✓			✓		3 (50)	
A01	8.5	✓	✓			✓		3 (50)	
A20	7	✓	✓	✓				3 (50)	
A21	6.5	✓	✓	✓				3 (50)	
A22	5.5	✓	✓	✓				3 (50)	
A11	9					✓		1 (17)	

^a^The articles are ordered vertically by the percentage of items that are covered per article. The items are ordered horizontally by the percentage of items that are covered by a particular article. The quality score for each article is also shown in the second column.

#### Item 4.1: Performance Metrics Used to Evaluate the Model

Contextually appropriate performance metrics and their parameters should be used and reported. For example, a decision threshold needs to be reported for classification performance metrics that are dependent on a threshold, such as when a probability is predicted and sensitivity is computed from that. Alternatively, the Brier score can be used for evaluating the goodness of estimated probabilities. Multiple metrics should be reported to provide a more complete understanding of model performance under different use scenarios.

#### Item 4.2: The Cost or Consequence of Errors

Discuss the consequential effect of potential errors made by an ML model. We encourage the use of specific performance metrics designed to support the discussion. For instance, the false-negative rate of a diagnostic model may result in a missed diagnosis, which may be detrimental for some patients. Similarly, the false-positive rate of a prognostic model may lead to subjecting some patients to potentially intrusive, let alone unsafe, interventions unnecessarily. Cost curves [75] offer a comprehensive analysis for optimizing the operating conditions of a classifier and can also be used for regression models.

#### Item 4.3: The Results of Internal Validation

The results of the error estimation evaluations on all of the metrics should be reported. The results should be reported on the training and test data sets, where applicable. If multiple modeling techniques or models are being compared, appropriate statistical comparisons should be reported.

#### Item 4.4: The Final Model Hyperparameters

The hyperparameters of the final model that will be used in practice should be reported, even if they are the default values of the analytics software used.

#### Item 4.5: Model Evaluation on an External Data Set

An external data set can be a hold-out from the original sample used in the study, although that would be a weak form of external validation. A data set from the same facility at a later point in time or from a different facility or facilities would provide stronger external validation. The performance metrics from internal and external validation should be compared.

#### Item 4.6: Characteristics Relevant for Detecting Data Shift and Drift

Data distributions change over time that may negatively affect model performance in the future or in different settings [76]. To be able to detect data shifts and drifts, a baseline characterization of the training population and decision-making processes is needed. Items 1.3 (current practices) and 2.4 (data characteristics) may be sufficient, with the evaluation results interpreted within that context. However, any additional data and process details that may be relevant for future use and deployment should be provided. For example, the performance of a model may be dependent on a particular pattern of care or specific referral criteria at a facility, and this should be specified if the dependence is known a priori.

### Category 5: Model Explainability

#### Overview

Reporting on items in this category aims to substantiate the ease or difficulty in which a human is able to comprehend how the proposed model produces its output [77,78]. This will demonstrate the comprehensibility or the lack thereof of factors that drive the decision-making process in the proposed model and can potentially enhance the model’s credibility and trustworthiness. A mapping of the category 5 items to the articles is presented in Table 8.

**Table 8:** Articles that discuss reporting items in category 5 (model explainability; n=17 articles)^a^.

Articles	Quality score	Reporting items				Total (n=3 items), n (%)
		5.3 (n=8, 47%)	5.1 (n=6, 35%)	5.2 (n=6, 35%)		
A21	6.5	✓	✓	✓	3 (100)	
A11	9	✓		✓	2 (67)	
A18a+b	9		✓	✓	2 (67)	
A01	8.5	✓		✓	2 (67)	
A26	8	✓	✓		2 (67)	
A20	7		✓	✓	2 (67)	
A03	5.5	✓		✓	2 (67)	
A13	9	✓			1 (33)	
A19	7		✓		1 (33)	
A10	5.5	✓			1 (33)	
A16	5.5		✓		1 (33)	
A22	5.5	✓			1 (33)	
A27	8.5				0 (0)	
A08	7				0 (0)	
A23	6				0 (0)	
A24	6				0 (0)	
A06	5.5				0 (0)	

^a^The articles are ordered vertically by the percentage of items that are covered per article. The items are ordered horizontally by the percentage of items that are covered by a particular article. The quality score for each article is also shown in the second column.

#### Item 5.1: The Most Important Features and How They Relate to the Outcomes

An important aspect of explainability for prognostic and diagnostic tools is feature importance. Describe the methods used to determine feature importance and document how the final ranking of features and their scores (where relevant) are determined. Furthermore, discuss the relative or absolute impact of each feature on the outcomes and, if possible, the functional form of this relationship. Common methods include Shapley Additive Explanations and partial dependence plots.

#### Item 5.2: Plausibility of Model Outputs

The resulting model outputs need to be clinically plausible according to domain experts and consistent with the current understanding. This can be demonstrated with reference to existing literature or by validation from domain experts. Explanations for deviations should be provided.

#### Item 5.3: Interpretation of the Model’s Results by an End User

Reporting how knowledge is communicated will help define the complexity of the interaction between domain experts and the ML model, which in turn will promote the acceptance of the proposed model. For example, when predicting whether a patient will develop complications in the first 48 hours after lung resection surgery, the model can be made to present calibrated probabilities along with a simple calculation of CIs, which are commonly used and understood by surgeons to assess risk.

### Coverage of Reporting Items

**Figure 2:**
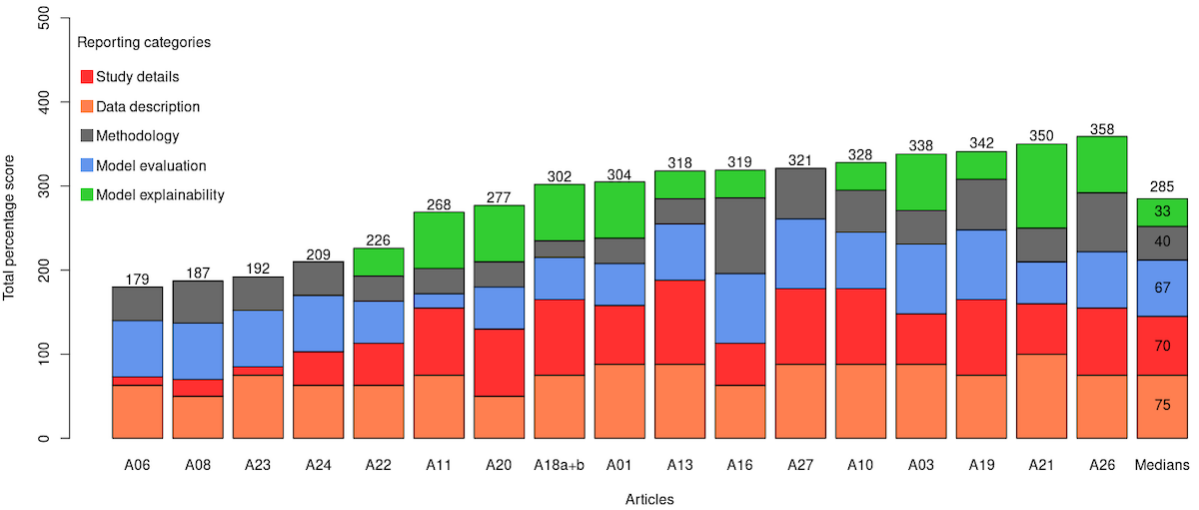
The coverage of reporting items and categories in each high-quality article. A bar (labeled with A##) represents an article and shows the proportions (%) of reporting items discussed in each category. The total score is calculated as the sum of all 5 proportions (out of 500). The medians in the right bar show the median proportions of proposed reporting items that were discussed in the consolidated high-quality articles.

We examined the coverage of the reporting items in each article in each of the reporting categories. Figure 2 presents the proportions of reporting items discussed in each article. The last bar on the right shows the median proportions of proposed items that were discussed in the articles for each category. Although the consolidated items have a higher focus on describing the “study details” (median 70%) and “data description” (median 75%), reporting on model explainability and details of methodology are lacking with a median percentage of 33% and 40%, respectively, being covered in those articles.

We also saw that some source articles, such as A06, A08, A23, and A24, did not cover all 5 categories that we defined. This demonstrates that the coverage of reporting items and categories is not uniform across source articles.

**Figure 3:**
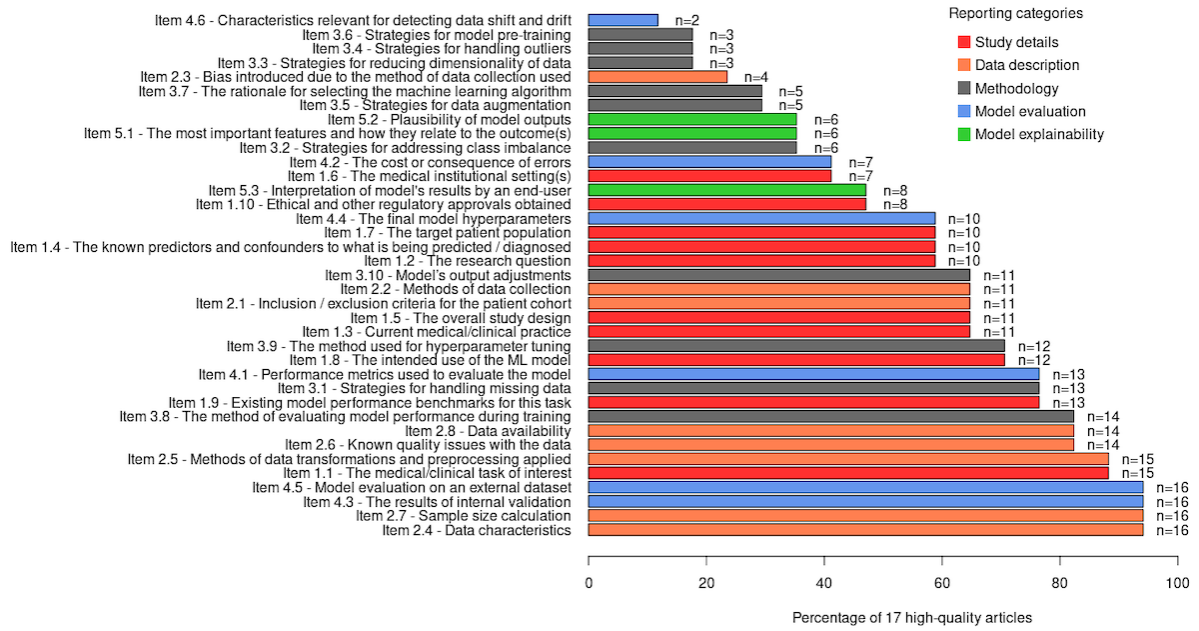
The list of reporting items, their categories, and their coverage in the source articles.

We also examined how often reporting items in this paper were discussed in the 17 high-quality articles. Figure 3 shows the list of reporting items and the number of articles that discuss them. We can see that some reporting items shown toward the top are rarely discussed and, not surprisingly, belong to the methodology category. Handling missing data and model performance evaluation during training are the most common methodology items.

The bottom of the figure with the items appearing in most articles is dominated by data description items. Sample size calculations and providing information on data characteristics are the most common in that category. Internal and external model evaluation items also appeared in almost all the articles. This is not surprising, because that is a basic requirement for in-silico studies.

Table 9 shows the rank correlation between article coverage and article quality score for the 17 articles. We can see that there is a strong and significant positive relationship only for the category “study details.” This suggests that the highest-quality source papers focused more on defining reporting items for the study details compared with articles with quality scores closer to the median. Coverage of the reporting items was not associated with article quality for the other categories, although the negative association for the “methodology” and “model evaluation” categories suggests that the highest-quality source papers focused less on those items.

**Table 9:** Spearman correlation between article coverage and quality score.

Category	Spearman correlation, ρ	*P* value
Study details	0.596	.01
Data description	0.23	.37
Methodology	−0.353	.16
Model evaluation	−0.348	.17
Model explainability	0.286	.27

## Discussion

### Summary

Multiple guidelines have been developed for ML prognostic and diagnostic modeling studies [3,4,27,45,46,50,52-59,61,62,79]. However, having multiple and overlapping guidelines can be confusing for researchers, journal editors, and reviewers, as it is less obvious which guidelines should be adhered to, and this may create friction in the adoption of good reporting practices. Such a state of affairs is not desirable, as there is evidence that the application of reporting guidance has benefits in higher-quality research and positive editorial decisions and increased citations.

In this paper, we developed a consolidated list of 37 reporting items for ML prognostic and diagnostic modeling studies conducted in silico or in shadow mode. The consolidated items were obtained from 17 high-quality source articles and represented consensus and expert guidance to ensure that these types of ML studies are adequately reported upon. The items were reviewed by independent experts and were applied in a review of a sample of *JMIR AI* articles, both of which informed further refinements and clarifications.

The results in Figure 2 support the need for our consolidated reporting items and checklist, since none of the source articles covered all 37 items presented. Some of our reporting items were covered in only 2 source articles, with the highest coverage being reporting items that appeared in 16 of the 17 source articles.

It was found that descriptions of the data and reporting study details were the most common categories in the source literature, whereas model explainability and methodological guidance (ie, data preparation descriptions and descriptions of model training) had the least coverage in the ML reporting guidelines literature overall. Using our checklist can help ensure that explainability and methodological considerations are documented appropriately. However, current explainability methods have come under criticism in terms of their value and the guarantees that they can provide [80].

The items can be used as a checklist (see the checklist in Multimedia Appendix 1), where the authors can indicate whether they have reported the relevant information. If it is reported, then the location in the article can be provided. If an item is not applicable, some reasoning can be provided. The use of a checklist will ensure consistency and that authors consider all reporting items.

Satisfying the reporting items does not necessarily mean that prognostic and diagnostic ML modeling study articles will increase in length. Some of the information may already be published elsewhere; therefore, the reporting items can be satisfied by citing other work. Any additional burden on authors will arguably be offset by the noted benefits to authors and readers, as well as by enhancing the transparency and reproducibility of ML studies.

### Limitations

Although we used a broad search of the literature to identify and select papers for our consolidated guidelines and checklist, we acknowledge that some may have been missed. However, any impact from missed papers is likely to be limited. In addition, a recent systematic review of reporting guidelines for prediction models that included ML within its scope did not identify any articles that were not within our scope [20].

Because this was a consolidation effort, whatever omissions exist in the literature, for example, will also be evident in our consolidated items. A design decision in this study was not to add new items that were not directly derived from the 17 source articles to ensure that we do not introduce a new source of bias in the results.

While we accounted for interrater reliability in the assessment of source article quality, the process of consolidating guidelines does involve subjectivity in the application of the inclusion and exclusion criteria. The external review by the *JMIR AI* editorial board helped ensure face validity to ameliorate some individual subjectivity.

The scope of our reporting items has been on structured data. ML modeling for other data types, such as text and images, was outside the scope of this study. Although this limitation excludes many studies, a large number of studies are still covered by our reporting guidelines and checklist. As noted earlier, approximately one-fifth of the noneditorial papers published in *JMIR AI* were on structured data. Future work should extend these consolidated reporting items and checklist to other data modalities (such as text and images).

Another scoping decision for the reporting items in this paper is that they focused only on in-silico and shadow mode studies. These are necessary first steps in developing and validating ML models. Future research can extend this work and cover the deployment and monitoring of prognostic and diagnostic ML models into practice.

This paper does not provide an explanation and elaboration for each reporting item. This information and references to examples are available in the source articles, as indicated in the mapping tables, and were therefore not repeated here.

The checklist that is provided in this paper (Multimedia Appendix 1) is intended to be used by authors to ensure that all relevant information for prognostic and diagnostic studies is reported. Extending the reporting checklist to an evaluation instrument would require additional effort to develop scoring items and scoring schemes [81,82]. This would allow the community to assess and track changes in reporting quality over time and evaluate study quality in meta-analysis projects.

## Appendix

Multimedia Appendix 1 Author checklist.

## Abbreviations

CONSORT-AI: Consolidated Standards of Reporting Trials–Artificial Intelligence
DECIDE-AI: Developmental and Exploratory Clinical Investigations of Decision support systems driven by Artificial Intelligence
ML: machine learning
PRISMA: Preferred Reporting Items for Systematic Reviews and Meta-Analyses
SPIRIT-AI: Standard Protocol Items: Recommendations for Interventional Trials–Artificial Intelligence
